# High Doses of Colchicine Act As “Silver Bullets” Against Severe COVID-19

**DOI:** 10.7759/cureus.54441

**Published:** 2024-02-19

**Authors:** Alexander Lilov, Kiril Palaveev, Vanyo Mitev

**Affiliations:** 1 Pulmonology, Specialized Hospital for Active Treatment Pneumo-Pneumonia-Phthisiatric Diseases (SHATPPD) “Sofia District” Hospital, Sofia, BGR; 2 Chemistry and Biochemistry, Medical University of Sofia, Sofia, BGR

**Keywords:** obesity, survival, colchicine, nlrp3 inflammasome, covid-19

## Abstract

A 48-year-old patient with a weight of 120 kg with type 2 diabetes mellitus, hypertension, and gout was hospitalized on the third day of the COVID-19 diagnosis. His general condition is relatively good, oxygen saturation is 89%. Despite starting standard treatment, on the seventh day from the onset of symptoms, the patient deteriorated sharply (oxygen saturation dropped to 74%). The negative development of the disease is interrupted with a loading dose of colchicine of 6 mg. This is a typical case of the life-saving effect of high but safe doses of colchicine in high-risk COVID-19 patients.

## Introduction

It is well established that obesity increases the risk of cancer, insulin resistance or type 2 diabetes, hypertension, atherogenic dyslipidemia, heart and other diseases [1]. According to the World Obesity Federation (2020), there are around 770 million adults globally affected by obesity, and one billion people globally are estimated to be living with obesity by 2030 [2]. About 2.6 billion people globally - 38% of the world population - are already overweight (BMI of 25) or obese (BMI is at least 30) [3]. According to 2017-2018 data from the National Health and Nutrition Examination Survey (NHANES), nearly one in three adults (30.7%) is overweight, more than two in five adults (42.4%) have obesity and about one in 11 adults (9.2%) have severe obesity (BMI above 40) [4].

Many studies have reported increased rates of hospitalization, mechanical ventilation, and mortality in COVID-19 patients with severe obesity [5-7]. Obesity prevalence demonstrates a positive and strong association with COVID-19 case rate and mortality. For every 1% increase in obesity prevalence, the case rate was higher by 6.6% and the mortality rate was increased by 8.3% [8]. Treatment of severely obese patients is a serious therapeutic problem, and solving it would significantly reduce COVID-19 mortality.

Here, we describe a case of a patient with severe obesity and multiple comorbidities who, despite standard hospital treatment, deteriorated sharply on day 7 of the onset of COVID-19. His life was saved as a result of the included treatment with high doses of colchicine.

## Case presentation

It concerns a patient, 48-year-old, hospitalized on October 31, 2021, to the ward with complaints for about 3-4 days of weariness, loss of appetite, easy fatigue at usual physical efforts, and measured high body temperature of 39°C, more profuse sweating, chills, cough with expectoration of white-colored sputum, feeling of shortness of breath. Due to these complaints he sought medical help, a positive antigen test for SARS-CoV-2, and started oral therapy with cephalosporin antibiotics, but due to lack of therapeutic effects after radiography, he was referred for hospitalization for conducting of therapy.

The patient was in mildly impaired general condition. Skin and visible mucous membranes were with normal coloration. Respiratory rate was 23/min with bilateral vesicular breathing, with single small moist wheezes to the left axillary. The heart activity was rhythmic with a frequency of 98 beats/min and blood pressure was 140/90 mm/Hg. The abdomen was soft, respiratory mobile, and painless at palpation. The liver and spleen were not found to be enlarged at palpation. The patient was a non-smoker and did not report allergies.

The patient reported the following concomitant diseases: Diabetes mellitus on oral therapy with Jentadueto 2.5 mg/1000 mg therapy; hypertensive disease on Nebilet 2x5 mg therapy; gout - currently without therapy; BMI was 46.8 kg/m^2^.

Laboratory blood test results from hospitalization to discharge of the patient are presented in Table 1.

**Table 1:** Laboratory blood test results during hospitalization ESR: erythrocyte sedimentation rate; Hb: hemoglobin; Leu: leucocytes; CRP: C-reactive protein; ASAT: aspartate aminotransferase; ALAT: alanine aminotransferase

Date	ESR	Hb	Leu	CRP	Ferritin	D-dimer	ASAT	ALAT
30-10-2021		146	9.5	67	2025	302		
01-11-2021	58	139	10.1			10000	59	68
05-11-2021	65	138	22.0	53.3		10000	18	55
11-11-2021	7	145	18.4				15	60
18-11-2021	6	160	29.0				17	41
29-11-2021	55	127	12.9	29.2	1675.56		43	191
Reference values	0-18 mm/h	130-180 g/l	3.5-10 x 10^9^/l	<0.5 mg/dl	30-400 ng/ml	0-500 mg/ml	0-42 U/l	0-40 U/l

From the tests performed on November 1, 2021 in the hospital were found also Ery-4.92 x 1012/l; Try-196 x 109/l; Gran-6.9 x 109/l; Lym-2.8 x 109/l; Mo: 0.4 x 109/l; glucose: 6.0 mmol/l; creatinine: 62 µmol/l.

The data of arterial blood gas analysis is represented in Table 2.

**Table 2:** Results of blood gas analysis during hospitalization

Date	pH	pCO_2_ (mmHg)	pO_2_ (mmHg)	HCO_3_ (mmol/l)	Sat O_2_ (%)
31-10-2021	7.54	24.7	49	22.3	89
03-11-2021	7.53	26.5	34.6	22.3	74
05-11-2021	7.49	27.1	47.8	20.9	86.5
08-11-2021	7.49	26.6	51.6	20.6	89
18-11-2021	7.50	29.3	77.8	23.4	95.9
22-11-2021	7.51	30.6	91	24.7	97
29-11-2021	7.45	25.4	100	22.4	99

Initial standard therapy included ciprinol 0.1 amp. 2x200 mg; clindamycin 0.6 amp. 3x600 mg; urbason 2x20 mg; fraxiparine 0.6; bromhexine hydrochloride 3x1 amp. (12 mg) inhalation and oxygen therapy with mask with volume expander at 10 l/min.

On November 3, 2021, due to worsening respiratory insufficiency against the background of ongoing medical and oxygen therapy, the patient was switched to noninvasive ventilation in spontaneous-timed (ST) mode with EPAP 7 mmH_2_O and IPAP 17 mmH_2_O at an inspiratory volume of 700 ml. The fraction of inspired O_2_ was 80%.

**Figure 1:**
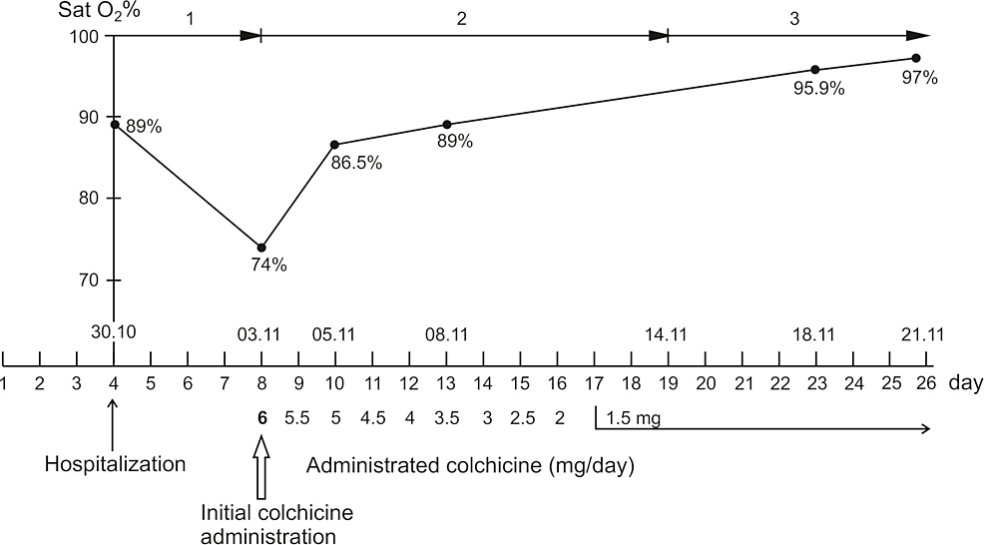
Dynamics of disease development before and after the administration of colchicine. Modes of ventilation: 1 - Mask with volume expander; 2 - Noninvasive ventilation in spontaneous-timed (ST) mode; 3- Noninvasive ventilation in continuous positive airway pressure (CPAP) mode.

The therapeutic regimen was also changed - the dose of urbason was increased to 2x100 mg, fraxiparine was discontinued and heparin therapy with perfusor at a dose of achieving control of 50-70 aPTT (activated partial thromboplastin time) was started. Novphyllin (aminophylline) 2x120mg IV, hymecromone 3x2 tabls., famotidine 2x1 amp (0.02g) IV, and degan 3x1 tabls were included. Colchicine was administered every 2 hours one tablet until reaching 12 tabs. (6 mg loading dose) then the dose was reduced by one tabl./daily until reaching 1.5 mg/day (supporting dose up to day 30) (Figure 1). Antibiotic therapy remained unchanged.

On the second day after the inclusion of colchicine (Table 2: 5-11-2021) a reduction of subjective symptomatology and a reduction of objective findings were registered.

Due to the betterment of the patient on November 14, 2021, ventilation was switched to noninvasive mode СРАР-7 mm/Н_2_О FiO_2_ 80 %, at Sat O_2_ 95%. On November 21, 2021, the noninvasive ventilation therapy was stopped and O_2_ therapy was started with a volume expander mask at 9 l/min and Sat O_2_ 94%. This therapy continued until December 3, 2021, after which O_2_ therapy was switched to nasal cannula therapy at 3 l/min. at Sat O_2_ 96%.

**Figure 2:**
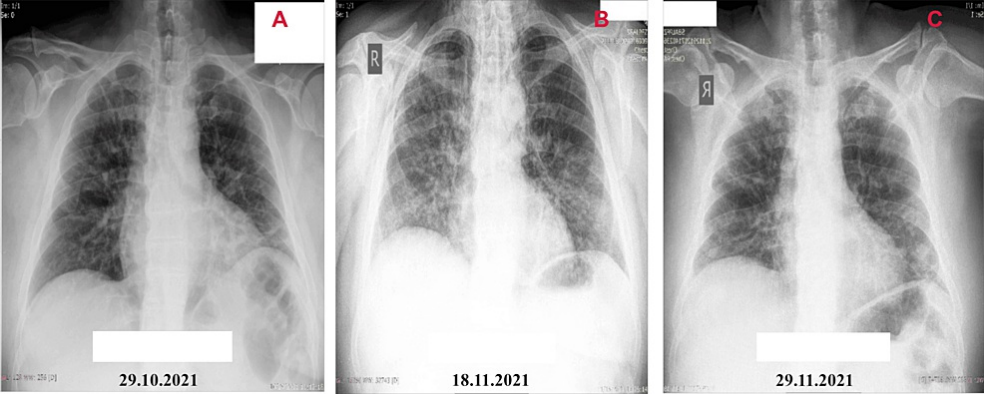
Radiography of lungs and heart conducted during the stay in the hospital А) Bilateral diffuse interstitial changes; В) Bilateral interstitial changes are more pronounced, affecting almost all lung fields; С) Bilateral interstitial changes are reduced compared to В.

Due to the onset of muscle atrophy of the limbs, probably also related to hypoglycemia, on November 20, 2021, after getting up, the patient collapsed, sustaining trauma in the gluteus area, as well as distortion of the knee joint (consultation with an orthopedist done), with hematoma forming a few days later, and on November 29, 2021, with abscess formation accompanied by subfebrility, consultation with a surgeon carried out (recommended daily medical treatment of abscess and hygiene). Radiography of lungs and heart conducted during patients' stay in hospital are presented in Figure 2. On November 30, 2021, the abscess cavity spontaneously drained, after which the symptoms of subfebrility were controlled and the edema significantly reduced. Microbiological examination of wound swab isolated Escherichia coli. AB therapy with gentamycin and metronidazole was started. On December 5, 2021, febrility again and severe scrotal pain with the formation of necrotic area on the skin for which he was referred for septic surgery where after two surgeries performed, the patient was discharged.

After discharge, the patient took colchicine 2x1 tabl. (1 mg/day) for 6 months.

**Figure 3:**
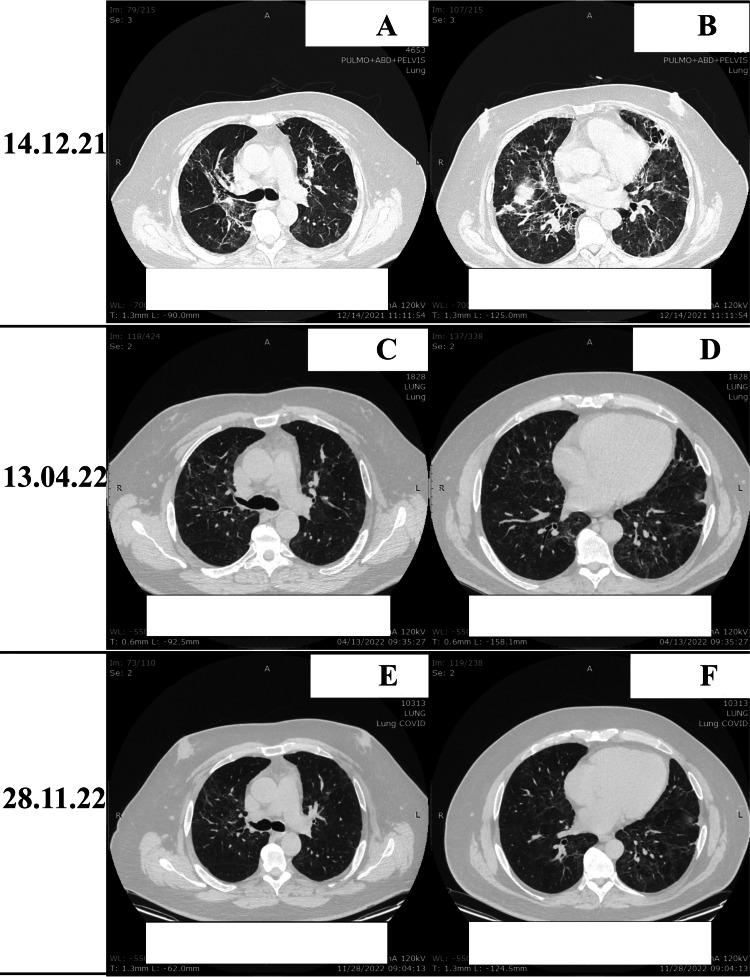
Control CT of lung А,B) Gross reticular changes with single and areas of consolidation bilaterally in the parenchyma; C,D) Still persistent reticular changes, single areas of consolidation peripherally; E,F) Reduction in the area of post-inflammatory changes associated with SARS-CoV-2.

Control computer tomography (CT) images of the lungs of the patient after discharge are presented in Figure 3.

## Discussion

The characteristic of this patient's treatment is that it proceeds in two stages. He was hospitalized on the third/fourth day of the onset of the disease in relatively good general condition. He was on standard COVID-19 treatment, which is administered according to the recommendations of the Ministry of Health. However, on the eighth day, the patient deteriorated sharply and his saturation dropped to 74% (Figure 1). Typically, such overweight patients with multiple comorbidities have a poor prognosis.

The second stage of treatment begins with the inclusion of 6 mg of colchicine, the dose of which is gradually reduced by 0.5 mg daily. To prevent a possible hyaluronan storm, we also included hymecromone. This type of treatment is successfully applied in four hospitals in Bulgaria [9-12]. In early 2023 we published results from the height of the SARS-CoV-2 epidemic that in 452 COVID-19 inpatients higher colchicine doses reduced the mortality about five times [11]. Analysis of another 333 cases of COVID-19 inpatients treated between 2020-2022, with increasing doses of colchicine, showed a clear trend of a reduction in mortality between 2- and 7-fold is submitted for publication.

The theoretical basis of this type of therapy is based on the following generally accepted postulates. The leading cause of death in COVID-19 is the cytokine storm, which is caused by an NLRP3 inflammasome hyperreaction [13,14]. Thus, our therapeutic strategy is based on inhibition of SARS-CoV-2 entry into the cell (bromhexine hydrochloride), inhibition of the NLRP3 inflammasome (with higher doses of colchicine), and the blocking of a possible hyaluronan storm with hymecromone [9,12].

Bromhexine is most effective when given prophylactically or started by inhalation after contact with a person with COVID-19. When the first symptoms of COVID-19 appear, the viral load is already at its maximum, which limits the effect of bromhexine [15]. Its earliest possible application is crucial for its effect [11,12].

Colchicine is known to block the NLRP3 inflammasome in vitro, however, attempts by dozens of clinical trials with low-dose colchicine to treat COVID-19 have been rather discouraging [16,17]. In 2022 and 2023, we published a series of articles and clinical cases in which we demonstrated the decisive effect of COVID-19 treatment with high doses of colchicine, justified the theoretical basis of its effect, and analyzed its safety [9-12,18].

Increased doses of colchicine are necessary for COVID-19 treatment due to the fact that it has the remarkable property of accumulating in leukocytes, and this leads to inhibition of NLRP3 inflammasome [19]. Thus, whereas plasma concentration after a single dosing of 0.6-mg colchicine is up to 3 nmol/l, it has been shown to accumulate in a saturable manner in neutrophils between 40 and 200 nmol/l. This means that at 6 mg of colchicine, its concentration in leukocytes can reach micromolar amounts sufficient to inhibit the NLRP3 inflammasome [12,18]. This also explains why the application of low doses of colchicine leads to rather discouraging results [17]. It is surprising that of all clinical trials with colchicine, only one investigated the effect of two different doses - low (1.6 mg) and high (4.8 for 6 hours), but this was for the treatment of a gout attack [12]. Another explanation for the conflicting results is that neither study took patient weight into account. It is not the same as giving a loading dose of 2 mg to a 50-kg patient and a 100-kg patient. The fact that obese patients have higher mortality in COVID-19 infection than non-obese patients, clearly states that the result of treatment with colchicine is closely related to the dose per kg of body weight [5-7,12]. The high doses we administer have been given widely in the past and are completely safe [9,11,18,20].

In comparison, the WHO-recommended paxlovid, remdesivir, and molnupiravir block viral replication. Since there is no direct link between viral replication and hyperactivation of the inflammasome, they are partially successful, with numerous side effects, incl. death, and besides, very expensive [12].

Blocking the hyperactivated NLRP3 inflammasome makes it unnecessary to use other very expensive drugs recommended for severe COVID-19, such as antibodies against the interleukin-6 receptor (tocilizumab and sarilumab) and Janus kinase (JAK) inhibitor baricitinib [12].

Recommendations

We recommend that any at-risk COVID-19 outpatient start immediate treatment with colchicine according to our regimen. This will prevent a possible cytokine storm and hospitalization. Every hospitalized patient must take immediately colchicine to avoid further complications, intubation, and death.

## Conclusions

This and other publications of ours demonstrate that high doses of colchicine can reverse the malignant course of COVID-19 and save the patient. Based on 3 years of our experience we believe that higher doses of colchicine are truly “silver bullets” against COVID-19 “werewolf”.
